# Arbitrary thickness profile metrology of low-*Z*and monolithic material components with a single X-ray projection

**DOI:** 10.1107/S1600577525005521

**Published:** 2025-07-31

**Authors:** Wenjie Hao, Feixiang Wang, Fucheng Yu, Kang Du, Ke Li, Junxiong Fang, Tiqiao Xiao

**Affiliations:** a Shanghai Institute of Applied Physics Chinese Academy of Sciences Shanghai201800 People’s Republic of China; b Shanghai Synchrotron Radiation Facility/Shanghai Advanced Research Institute Chinese Academy of Sciences Shanghai201204 People’s Republic of China; c University of Chinese Academy of Sciences Chinese Academy of Sciences Beijing100049 People’s Republic of China; University of Malaga, Spain

**Keywords:** X-ray phase contrast imaging, thickness metrology, single projection measurement, metrology of microlens array

## Abstract

Low-*Z* and monolithic material components with arbitrary thickness profiles are extensively utilized in heat conduction, biocompatible implants, microfluidics and integrated optics, where precise thickness measurement is crucial for quality control and performance analysis. X-ray micro-computed tomography (micro-CT) is widely employed for thickness metrology of such samples due to its nondestructive nature, high resolution and 3D imaging capabilities. However, the time-consuming projection acquisition and image reconstruction processes hinder it from efficient or dynamic thickness measurements. Additionally, micro-CT struggles with laminar samples. To overcome these limitations, we introduce X-ray phase contrast imaging for the thickness metrology of low-*Z* materials with arbitrary profiles by accurately retrieving the phase shift of X-rays passing through the sample from a single projection. Calibration using a standard nylon fiber demonstrates that within a 1.33 mm field of view (FOV) the method achieves a mean absolute error of 0.68 µm for cylindrical fibers with diameters of 407.14 µm. We further demonstrate the method’s capability for efficient measurement and damage assessment using a worn fiber with complex geometry. Additionally, we applied this method to the thickness measurement and error analysis of a microlens array with varying sub-lens parameters. The 3D profiles of all sub-lenses were obtained from a single projection, facilitating error analysis of height, symmetry and eccentricity. The results highlight the method’s advantages, including being *in situ*, non-contact and high precision, and having a large FOV, flexible adjustability and penetrative measurement capabilities. Our open device design suggests potential applications for dynamic thickness measurements and real-time monitoring of samples within *in situ* loading devices.

## Abstract 2

A thickness metrology method based on X-ray phase contrast imaging is proposed. By precisely retrieving the phase shift of X-rays passing through the sample, high-precision arbitrary thickness profile measurement can be achieved with a single X-ray projection. A measurement case of a microlens array is presented to demonstrate the effectiveness of this method.

## 1. Introduction

Micrometre-scale components fabricated from low-*Z* mater­ials with arbitrary thickness profiles are critical in various applications, including carbon thermal pads for heat dissipation in semiconductor circuit boards (Khan *et al.*, 2020 ▸), biocompatible implant devices (Mei *et al.*, 2022 ▸) and micrometre-scale optical components in integrated optics (Cai *et al.*, 2021 ▸). Thus, *in situ* non-destructive high-precision thickness measurements of these components are frequently required in quality control, performance analysis and manufacturing optimization (Budzik *et al.*, 2021 ▸). However, for such components with complex geometries, existing measurement methods often struggle to achieve simultaneous *in situ*, non-contact, high-precision and efficient measurements.

In X-ray-based thickness metrology, various methods have been developed for measuring the thickness of complex structured samples (Isomura *et al.*, 2019 ▸; Leri & Ravel, 2014 ▸). Among these, micro-computed tomography (micro-CT) has been extensively utilized for 3D reconstruction of samples, providing high-precision measurement and comprehensive analysis (Li *et al.*, 2020 ▸; Li *et al.*, 2021 ▸; Chen *et al.*, 2011 ▸). The unique advantage of X-ray-based metrology lies in its insensitivity to the sample’s orientation relative to the incident beam, allowing straightforward measurement of arbitrary profiles. Even enclosed samples can be measured, provided the container is X-ray permeable and its properties are known, thereby offering a distinct advantage for measuring components within *in situ* devices (Liu *et al.*, 2024 ▸). However, CT scanning and reconstruction processes are time-consuming and laminar samples are unsuitable for rotational scanning (Sun *et al.*, 2020 ▸). Consequently, we hope to reconstruct the thickness profile directly by analyzing information from X-ray projection images, which is undoubtedly a more efficient scheme and can be applied to real-time measurements of dynamic processes. For materials with measurable X-ray absorption, thickness can be determined based on the exponential relationship between thickness and intensity attenuation (Isomura *et al.*, 2021 ▸; Gójska *et al.*, 2024 ▸). However, this method is ineffective for thin and low-*Z* materials with imperceptible X-ray absorption. In comparison to intensity contrast, the phase shift in the transmitted X-ray wavefield induced by the component exhibits a more sensitive linear relationship with thickness (Momose, 2017 ▸), making it a superior solution for high-precision measurement of such components. Nonetheless, precise phase retrieval is a pre­requisite for utilizing X-ray phase shifts in thickness measurements. Recent advancements in high-quality X-ray sources (Xie *et al.*, 2020 ▸), coupled with progress in various phase retrieval methods (Glinz *et al.*, 2024 ▸; Zdora *et al.*, 2017 ▸; Olivo & Speller, 2007 ▸), have rendered this approach feasible.

Recently, the speckle-based phase contrast imaging (SBI) method has experienced rapid development due to its high spatial resolution, phase sensitivity and experimental flexibility (Morgan *et al.*, 2012 ▸; Berujon *et al.*, 2012 ▸). This method employs ordinary sandpaper as a phase modulator, providing an adaptable and cost-effective approach. SBI achieves phase-contrast and dark-field imaging by tracking speckle displacement and deformation (Zanette *et al.*, 2014 ▸). Wang *et al.* (2017 ▸) introduced a double-exposure method that significantly enhanced phase retrieval accuracy, and its effectiveness in micro-CT was subsequently demonstrated (Yu *et al.*, 2021 ▸). Later, Yu and co-workers reported the use of a modified neural network in the double-exposure method, enabling precise phase retrieval with a single projection (Yu *et al.*, 2024 ▸). Due to its efficient data acquisition and precise phase retrieval, this deep-learning-based double-exposure speckle tracking X-ray phase contrast imaging (DLD-STXI) method holds significant potential for X-ray metrology.

In this paper, we apply the DLD-STXI method to the thickness metrology of single-material samples with arbitrary profiles. We begin by outlining the principles and experimental setup of the method. A standard sample is employed to evaluate accuracy, followed by a dedicated sample to demonstrate the capability for handling complex thickness profiles. To assess the method’s applicability to actual devices, a microlens array is tested and quantified using the proposed approach. Finally, we conclude with a summary of our findings.

## 2. Method

### 2.1. Principle

**Figure 1:**
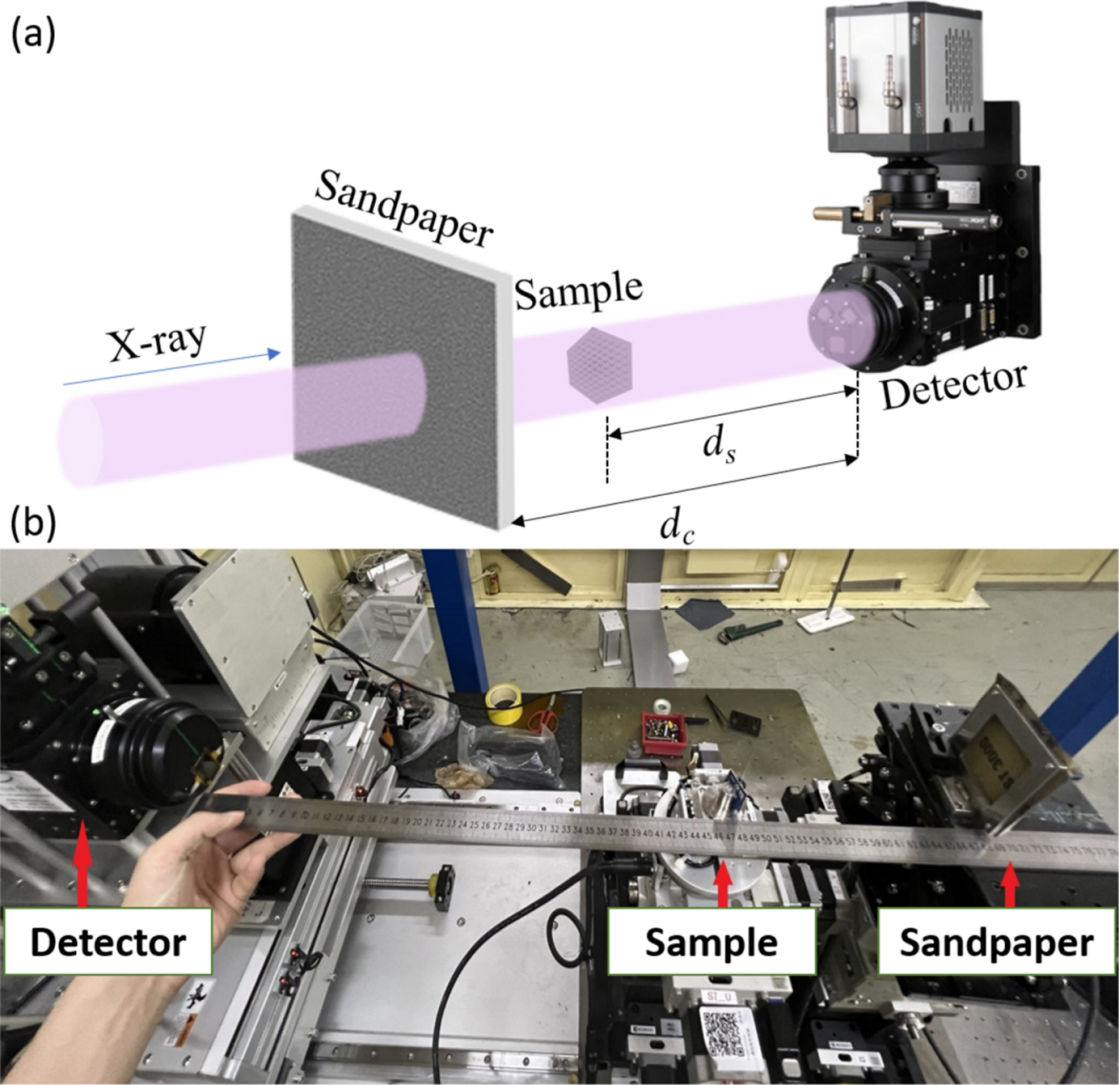
Experimental setup of the DLD-STXI method: (*a*) a schematic diagram and (*b*) a photograph of the facilities.

The setup of the DLD-STXI method for metrology is illustrated in Fig. 1 ▸(*a*). We use sandpaper, a common and readily available diffuser, to generate a speckle pattern. After propagating a certain distance marked *d*_c_, this pattern is recorded by a high-resolution detector in the Fresnel diffraction zone. We employ a pre-recording scheme, with the original speckle image captured before the measurement. As a result, the measurement efficiency is improved significantly.

In the experimental setup, the sample is positioned in the optical path downstream of the sandpaper and then modulated by the speckle pattern. The displacement and deformation of the speckles correlate with the phase and dark-field signals of the sample, respectively. To track these variations between the reference pattern and the distorted pattern at a sub-pixel level and achieve high-contrast imaging, we employ the digital image correlation (DIC) algorithm (Pan *et al.*, 2013 ▸; Wu *et al.*, 2024 ▸). Along the direction of light propagation on the *z* axis, the relationship between phase shift and speckle displacement is given as (Morgan *et al.*, 2012 ▸)
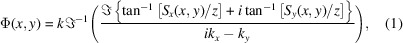
where the wavenumber *k* = 2π/λ, 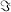 and 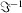 denote the Fourier transform and inverse Fourier transform, respectively, and *k*_*x*_ and *k*_*y*_ are the corresponding coordinates. The thickness of the sample along the *z* axis is linearly related to the phase shift, 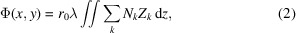
where *r*_0_ is the classical electron radius, λ is the wavelength, *N*_*k*_ is the number of atoms per unit volume for the element *k* and *Z*_*k*_ is the atomic number. For a homogeneous medium sample, *k* is uniquely determined, meaning it is a linear integration over the thickness in the *z* direction (Momose, 2017 ▸). The relationship between the deformation of the speckles and the dark-field signals *D*(*x*, *y*) can be expressed as (Wang *et al.*, 2016 ▸)
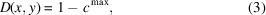
where *c*^max^ is the peak coefficient of the correlation algorithm.

The intensity distribution captured on the detector is formulated as (Wang *et al.*, 2017 ▸)
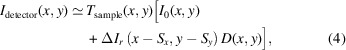
where *T*_sample_(*x*, *y*) denotes the sample’s transmission, which is in fact the in-line phase contrast image of the sample. *I*_0_(*x*, *y*) is the intensity of the incident X-ray beam, Δ*I*_*r*_(*x* − *S*_*x*_, *y* − *S*_*y*_) represents the speckle displacement and *D*(*x*, *y*) denotes the dark field. The double-exposure method addresses the issue of phase distortion by separately capturing the sample’s in-line phase contrast image under identical experimental conditions.

In speckle-based imaging (SBI), interface edge enhancement artifacts degrade demodulation accuracy. While the double-exposure method (Wang *et al.*, 2017 ▸) can remove this effect, it has three drawbacks: (i) it prevents dynamic measurements, (ii) it reduces efficiency and (iii) it requires error-prone image registration. Our DLD-STXI method uses a neural network based on the architecture of a conditional generative adversarial net (CGAN) to generate the in-line phase contrast image from a single speckle pattern, achieving double-exposure equivalence without its limitations. This is possible because the speckle is a kind of randomly distributed pattern, whereas the in-line phase contrast image has distinct structure. The different distribution patterns of these two types of composition allow the neural network to distinguish them. As a result, the thickness profile of the sample can be accurately reconstructed from just a single projection using our proposed technique. The relevant details are documented in our previous papers (Yu *et al.*, 2024 ▸; Du *et al.*, 2024 ▸).

### 2.2. Experimental setup and data processing

We conducted DLD-STXI-based metrology experiments on beamlines 13HB and 09B at the Shanghai Synchrotron Radiation Facility (SSRF). The experimental facilities are illustrated in Fig. 1 ▸(*b*).

For imaging-based metrology, the field of view (FOV) and resolution were adjustable to meet specific requirements. On beamline 13HB, high-resolution measurements were carried out utilizing a 15 keV monochromatic X-ray beam, with each exposure lasting 1.5 s. The detector (Hamamatsu ORCA-Flash 4.0, model C11440) was positioned 45 cm downstream from the seven-axis rotating stage (KOHZU, model RA04A-W01). After the scintillator (LuAG, Ce), a 10× magnifying lens (Optique Peter, model MICRX016) was used to transfer the image to an SCMOS camera with a pixel size of 6.5 µm, achieving an effective pixel size of 0.65 µm for the metrology system. On beamline 09B, a 2× magnifying lens was employed to achieve a wider FOV. Here, a 15 keV monochromatic X-ray beam was employed with a 2.5 s exposure time, and the detector, with an effective pixel size of 2.3 µm, was positioned 95 cm downstream of the sample. The sample-to-detector distance in these setups was carefully chosen to ensure sufficient propagation of the speckle displacement caused by the phase shift. As the propagation distance increases, the spatial displacement of the speckle patterns becomes more pronounced. With high-resolution detectors, this displacement can be observed at closer distances, while for lower-resolution detectors, a greater distance is required. However, this distance cannot be too long, as the DIC algorithm would then need to search over a larger area, imposing a heavy computational burden.

The first step before thickness metrology is to adjust the sample to be perpendicular to the incident X-ray beam. In the case of laminar samples, setting the normal of the planar object parallel to the optical axis is especially important. This ensures that the thickness information obtained is not stretched or compressed in any direction. We use a laser positioning device in conjunction with a high-precision seven-axis rotary stage to achieve precise alignment. Another practical method for verification is to observe the edge region of the laminar sample in absorption-based imaging to check for ghosting. If there is no ghosting, it indicates that the front and back planes of the sample are parallel with the projection plane and the sample is strictly perpendicular to the incident beam.

**Figure 2:**
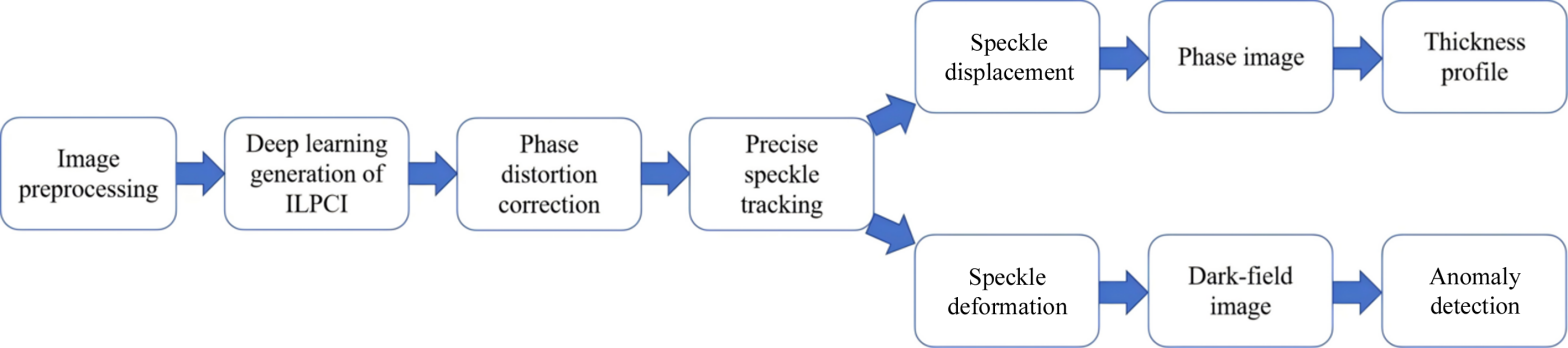
Flowchart for precise phase retrieval based on a single projection.

The workflow for phase retrieval based on a single projection is outlined in Fig. 2 ▸. The process involves image pre­processing, using a neural network to generate in-line phase contrast image (ILPCI) from the speckle image, eliminating the phase distortion effect with the double-exposure method, and applying the DIC algorithm to reconstruct phase and dark-field images. Finally, the precisely retrieved phase is used to obtain the thickness profile of the sample.

## 3. Results

### 3.1. Accuracy calibration with microtomography

To calibrate the accuracy of the proposed approach, we cut a uniform segment from a nylon fiber used as a high-strength weaving material, which was selected as the standard sample. All the data acquisitions were carried out on BL13HB at the SSRF using a detector with an effective pixel size of 0.65 µm. To isolate errors introduced by the imaging-based metrology from those caused by detector precision or other environmental factors, we conducted a micro-CT scan under identical conditions. For consistency, the same detector was used for both the CT and DLD-STXI measurements. In the DLD-STXI analysis, the DIC window size was set to 5 pixels with a step size of 1 and the search region was extended by 5 additional pixels. These parameters were selected to ensure that the effective pixel size in the DIC-based reconstruction matched that of the CT, enabling a fair comparison.

Due to the weak X-ray absorption of nylon, conventional absorption-based CT struggles to produce clear boundaries and sufficient contrast for such a low-*Z* material. Therefore, we did not use traditional CT in our calibration, but instead employed in-line phase-contrast CT (IL-PCI CT) based on the phase-attenuation duality algorithm (Chen *et al.*, 2012 ▸). This method enables accurate reconstruction of single-material samples with enhanced edge visibility and contrast, making it ideal for quantitative analysis of low-*Z* materials (Li *et al.*, 2020 ▸; Li *et al.*, 2021 ▸; Chen *et al.*, 2011 ▸). The IL-PCI CT reconstruction served as our ground truth and further details are provided in Appendix *A*[App appa]. These improvements ensure that the segmented results from IL-PCI CT offer a valid and accurate reference for evaluating the error in our proposed DLD-STXI method.

**Figure 3:**
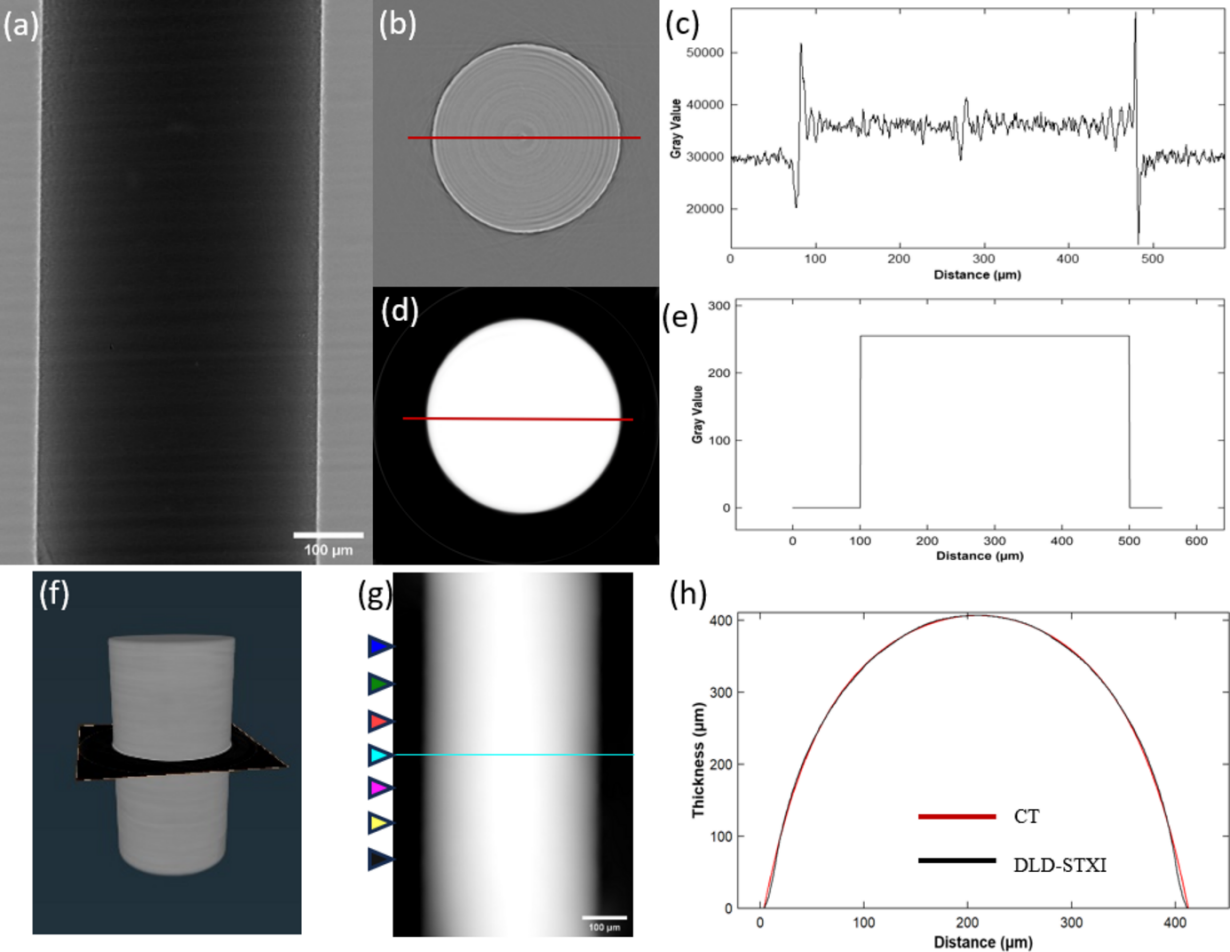
Thickness metrology of a nylon fiber with 0.65 µm pixel size. (*a*) Projection image, (*b*) CT slice from absorption-based CT, (*c*) corresponding profile of (*b*), (*d*) CT slice from IL-PCI CT, (*e*) binary contour extracted from (*d*), (*f*) 3D rendering of IL-PCI CT, (*g*) phase image from DLD-STXI and (*h*) thickness profiles from IL-PCI CT and DLD-STXI along the blue line in (*g*).

The CT slices and edge profiles obtained using conventional CT are shown in Figs. 3 ▸(*b*) and 3 ▸(*c*), where weak contrast and edge ambiguity are evident. In contrast, the IL-PCI CT result in Fig. 3 ▸(*d*) demonstrates significantly improved contrast and well defined edges, enabling the precise measurement of a 407 µm fiber diameter. Thresholding and binarization of this image yielded the final boundary profile shown in Fig. 3 ▸(*e*) and resulted in the 3D volume in Fig. 3 ▸(*f*). Both CT and IL-PCI CT [Fig. 3 ▸(*b*) and Fig. 3(*d*)] reconstructions were performed from 720 projections over a 180° rotation using the FBP algorithm (Burvall *et al.*, 2011 ▸). The thickness maps shown in Fig. 3 ▸(*h*) were derived by integrating voxel values along the projection direction. From Fig. 3 ▸(*h*), it is obvious that the two curves fit quite well, which confirms the high accuracy of the proposed DLD-STXI method for metrology of a thickness profile with a single planar projection.

**Figure 4:**
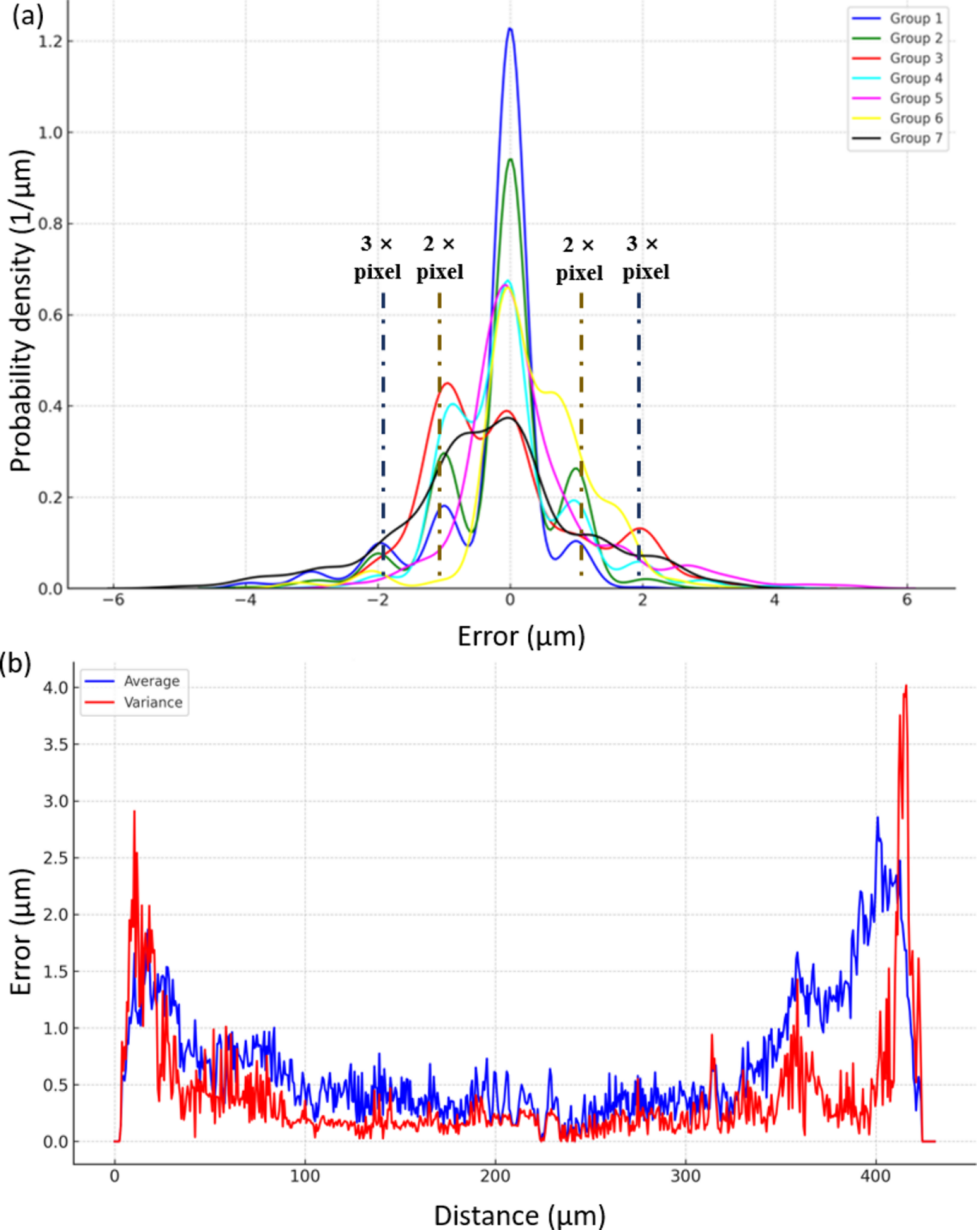
Statistical analysis of errors for the thickness profile measured by the proposed DLD-STXI method. (*a*) Probability density distribution of errors and (*b*) spatial distribution of the average and the variance of the absolute errors.

For a comprehensive evaluation of the performance of the DLD-STXI method, quantitative analysis on multiple sections of the sample was also carried out. As shown in Fig. 3 ▸(*g*), seven equally spaced sections were selected for further quantitative analysis, marked with different colors for the convenience of subsequent error analysis. The results of the quantitative analysis are given in Fig. 4 ▸. In Fig. 4 ▸(*a*), the normalized error density curve shows the probability density distribution of errors across multiple fiber sections, where each curve corresponds to a position indicated in Fig. 3 ▸(*g*). The vertical axis represents the probability density with units of µm^−1^ and the area under each curve integrates to 1. The compact nature of the curves indicates that the data points are concentrated within a smaller error range. An interesting phenomenon observed is the presence of a centrally symmetric multi-peak distribution in the curves, reminiscent of the light intensity distribution from the diffraction of a monochromatic point source. This phenomenon is particularly evident in the first, second and fourth groups of curves. It can be seen in the figure that the first peak, which is the highest, is located near zero, while the secondary peaks appear at ±1.3 µm. Given that the image was captured using a detector with an effective pixel size of 0.65 µm, 1.3 µm corresponds exactly to twice the pixel size. The tertiary peaks are observed near ±1.95 µm, which corresponds to three times the pixel size. This phenomenon suggests that pixel size is a significant factor affecting the error distribution. Similar to the definition of the Rayleigh criterion, we calculated the proportion of all data points with errors within twice the pixel size at 84.17%, indicating that the measurement accuracy has approached the resolution limit of the detector.

Fig. 4 ▸(*b*) shows the spatial distribution of the average and the variance of the absolute errors along the fiber, providing a clearer overall trend. The total mean absolute error (MAE) and root-mean-square error (RMSE) are calculated to be 0.683 µm and 1.033 µm, respectively. The average MAE value indicates that the average deviation from the ground truth is very close to the effective pixel size of 0.65 µm of the detector used for data acquisition. This demonstrates that the absolute error of the proposed method is largely determined by the effective pixel size of the detector. Overall, the results obtained using DLD-STXI with a single-shot image are comparable to those obtained through CT reconstruction using 720 projection images, highlighting the high accuracy and convenience of the DLD-STXI method. The average RMSE calculated is 1.033 µm, which exceeds the average MAE. As illustrated in Fig. 4 ▸(*b*), larger errors primarily cluster around the sample’s peripheries, characterized by regions with high spatial frequencies. Conversely, within the central area of the sample, error values are notably smaller with reduced fluctuations. Decreased measurement precision for high-frequency components poses a typical challenge in metrology, often influenced by sampling frequency, which in this method is represented by the detector pixel size. Additionally, discrepancies between neural network-generated in-line phase contrast images and actual measurements exacerbate these errors. This phenomenon suggests that, despite the widespread use of neural networks for feature extraction and image generation, further improvements in accuracy remain necessary. Encouragingly, deep learning has garnered considerable traction in the realm of quantitative imaging, and ongoing advancements by researchers (Qiao *et al.*, 2022 ▸; Shen *et al.*, 2023 ▸; Ge *et al.*, 2021 ▸; Zhu *et al.*, 2021 ▸) are anticipated to enhance algorithmic precision for image generation.

In summary, based on calibration with a standard sample and using micro-CT as the ground truth, the accuracy of the proposed method, represented by the MAE and RMSE, is 0.683 µm and 1.033 µm, respectively. The measured diameter of the sample is 407.14 ± 0.68 µm.

### 3.2. Metrology of samples with arbitrary thickness profiles

**Figure 5:**
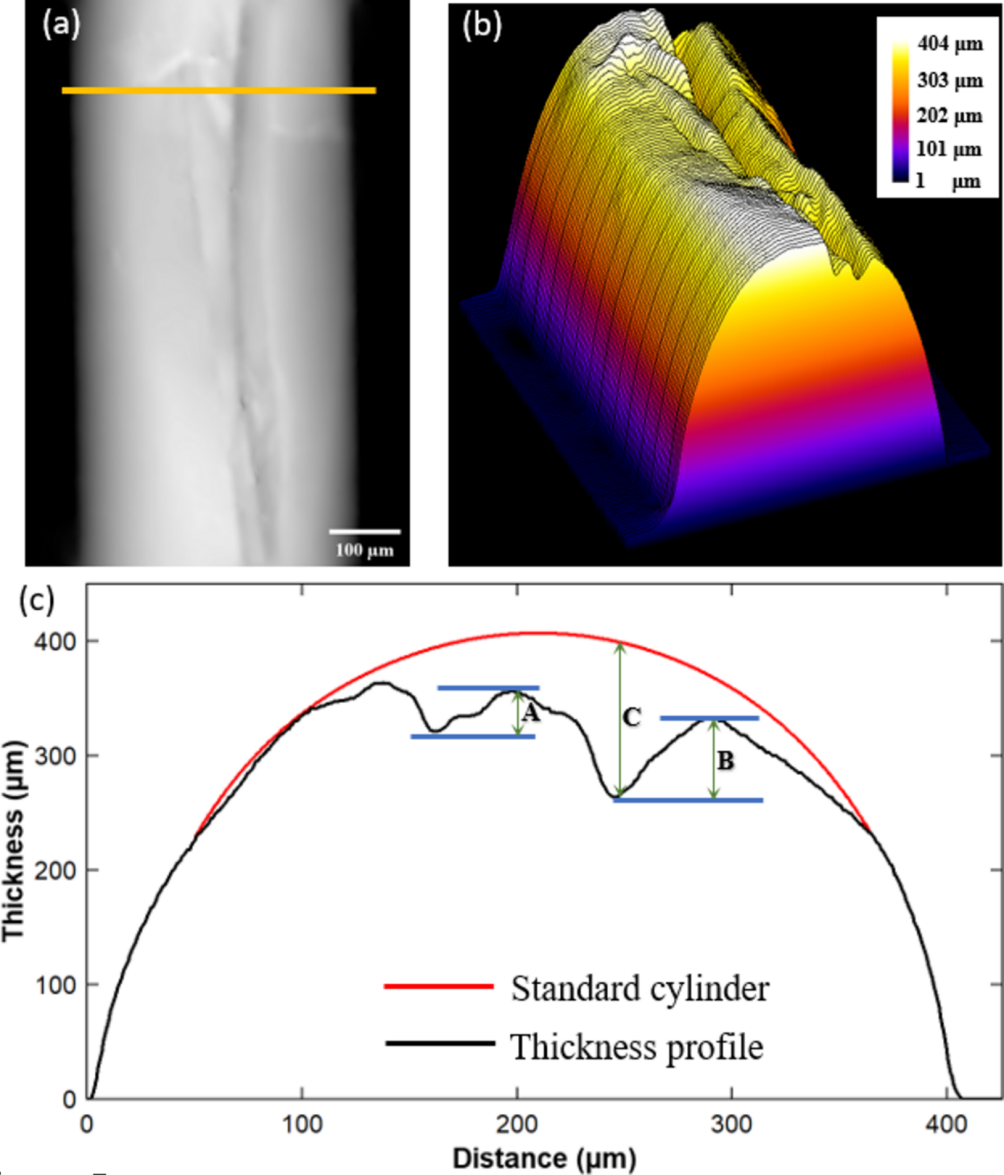
Measurement results on the worn-out fiber by DLD-STXI. (*a*) Phase image, (*b*) 3D thickness profile and (*c*) thickness profile along the section denoted with a yellow line in (*a*).

To evaluate the capability of the DLD-STXI method in measuring samples with arbitrary thickness profiles, we found a worn-out segment of the above-mentioned nylon fiber, which exhibited an irregular surface profile, as the test sample. The metrology was also conducted on beamline 13HB at the SSRF, with experimental conditions consistent with those described in the earlier section. The experimental results are shown in Fig. 5 ▸. Fig. 5 ▸(*a*) shows the phase image reconstructed by the DLD-STXI method, while Fig. 5 ▸(*b*) illustrates the corresponding 3D thickness profile based on the precisely retrieved phase information of the worn-out fiber. It is evident that the irregular thickness profile of the fiber is successfully revealed.

The curve in Fig. 5 ▸(*c*) delineates the thickness profile along the section denoted by the yellow line shown in Fig. 5 ▸(*a*). Two obvious scratches can be seen in Fig. 5 ▸(*c*) along the line, where the depths of scratch A and scratch B are 30.5 µm and 65.3 µm, respectively. Compared with the ideal profile of a standard cylinder (using the uniform fiber diameter data measured in the earlier section), the wear depth C is estimated to be 131.6 µm. In general, the test results of the worn-out fiber demonstrate that, for a total thickness of approximately 407 µm, scratch depths of 31–131 µm can be measured. According to the calibration given in Section 3.1, the average RMSE of the proposed method is around 1 µm. Thus, the proposed DLD-STXI method has a large dynamic range and high accuracy for the metrology of materials with arbitrary thickness profiles. The FOV of such an imaging metrology method is usually determined by the detector. In our case, the FOV of the detector is 1.33 mm, meaning that samples with sizes not larger than 1.33 mm can be effectively measured by the proposed method based on a single planar projection.

### 3.3. Metrology of a microlens array

To demonstrate the practical application of the proposed method, we applied it to a micrometre-scale optical component, a multi-focus microlens array fabricated via photopolymerization-based additive manufacturing. The metrology was carried out on the R&D beamline at the SSRF (BL09B) and the projections were collected by a detector with an effective pixel size of 2.3 µm. This array consists of 91 sub-lens units arranged in a hexagonal pattern, radiating from the center to the periphery, with each unit having a lens radius of 125 µm and uneven height profiles. The height of each lens decreases from the inside out. More details about the microlens array are presented in Appendix *B*[App appb].

**Figure 6:**
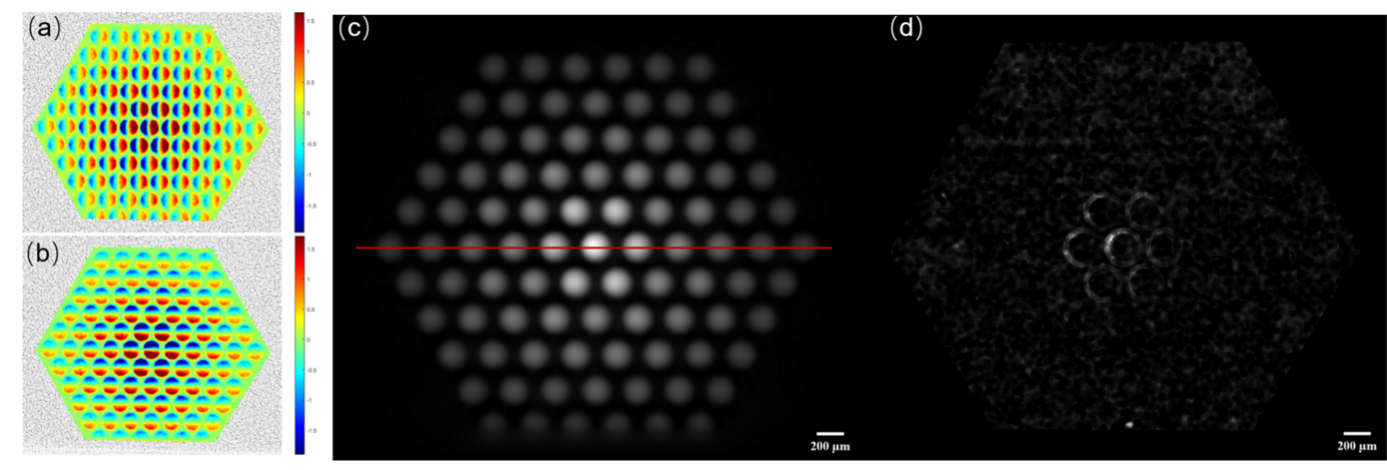
Imaging metrology of the micro-lens array by DLD-STXI. (*a*) The *u* displacement map of speckle tracking X-ray imaging, (*b*) the corresponding *v* displacement map, (*c*) phase image and (*d*) dark-field image.

Imaging-based metrology of the micro-lens array using DLD-STXI is shown in Fig. 6 ▸, where Figs. 6 ▸(*a*) and 6 ▸(*b*) display the displacement maps of speckles in the *u* and *v* directions, respectively. No obvious anomalies are found in the figures. Speckle displacement is highly sensitive to phase shift, with anomalies in the displacement map typically indicating roughness and unevenness. On the R&D beamline at the SSRF (BL09B), researchers have developed a high-sensitivity surface roughness of optical devices detection system based on this principle (Xue *et al.*, 2023 ▸). A phase image of the micro-lens array is shown in Fig. 6 ▸(*c*), and based on this phase distribution the thickness distribution of the micro-lens array is normal overall. However, according to the dark-field image shown in Fig. 6 ▸(*d*), the significant and asymmetric outline of the micro-lens in the central part of the lens array due to abnormal X-ray scattering indicates the presence of manufacturing errors. This means that further quantitative analysis on the thickness profile of all the lenses is required to evaluate the quality of the micro-lens array produced by additive manufacturing.

**Figure 7:**
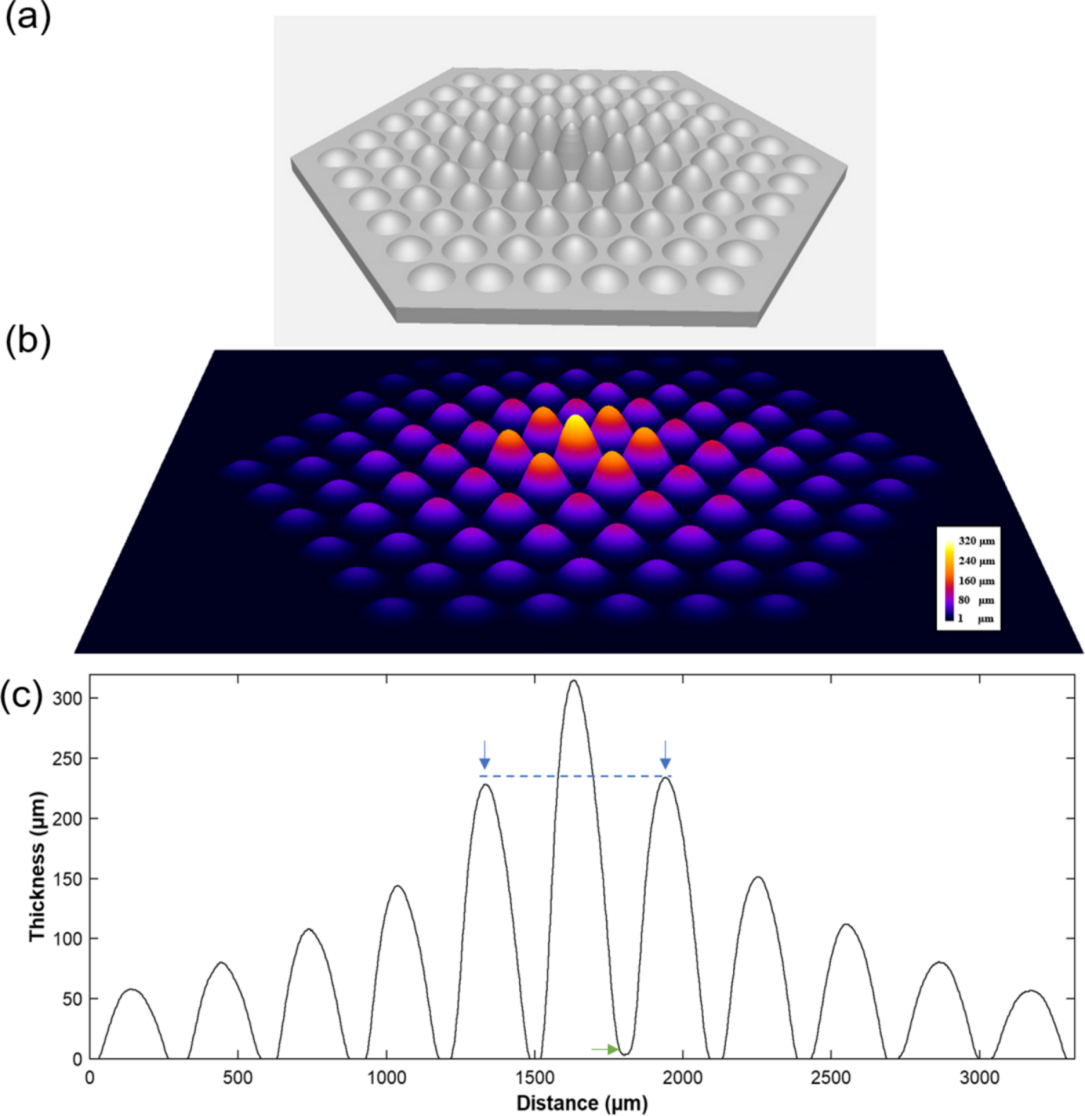
Thickness profile of the micro-lens array. (*a*) Design diagram, (*b*) 3D thickness profile and (*c*) thickness profile along the red line shown in Fig. 6(*c*).

Based on the phase information given in Fig. 6 ▸(*c*), the 3D thickness profile of the microlens array is illustrated in Fig. 7 ▸(*b*) while Fig. 7 ▸(*a*) provides the design diagram. A comparison between the sets of data reminds us of the existence of errors such as height discrepancies, tilting angles and symmetrical characteristics, which may adversely affect the optical performance. Here, a group of micro-lenses is selected for further quantitative analysis, as denoted by a red line in Fig. 6 ▸(*c*), and the thickness profiles of the lenses are shown in Fig. 7 ▸(*c*). According to Fig. 7 ▸(*c*), it is obvious that there is a toe protrusion on the right-hand side of the central lens, marked by a green arrow, and the height of the protrusion is about 3.5 µm. Accordingly, a height discrepancy between these two adjacent lenses is also observed, indicated by two blue arrows. Specifically, the height of the right-hand side lens is larger than its symmetrical counterpart, with a height difference of 5.8 µm.

**Figure 8:**
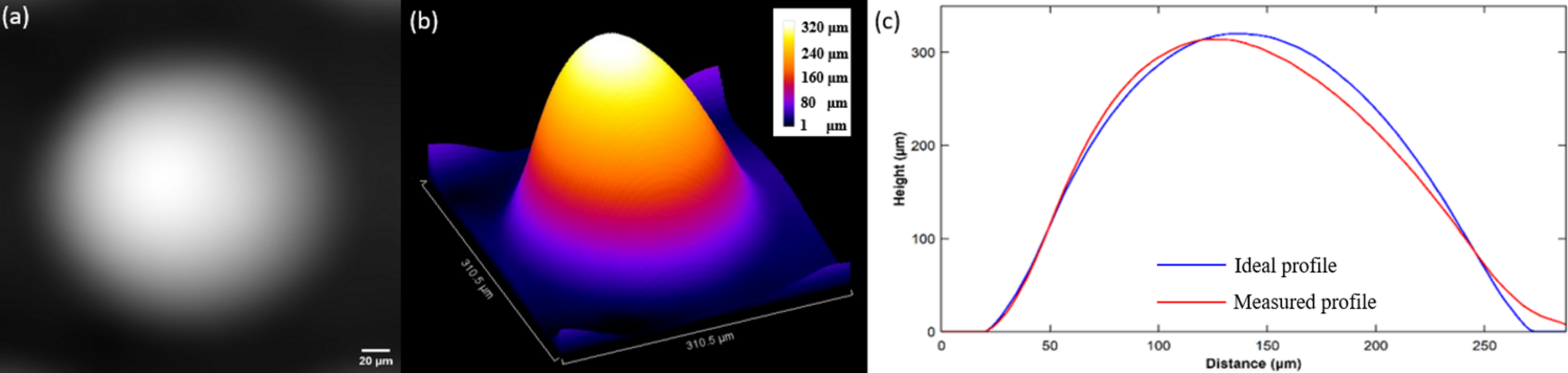
Metrology of a separate lens unit located in the center of the micro-lens array. (*a*) Phase image, (*b*) 3D thickness profile and (*c*) measured thickness distribution compared with the ideal profile.

As an example for the metrology of a separate lens unit, the central lens was selected for more detailed analysis and the results are shown in Fig. 8 ▸. Shown in Fig. 8 ▸(*a*) is the phase image of the lens unit retrieved by DLD-STXI. According to its grayscale distribution, an asymmetric thickness profile from the right to the left side of the lens can be observed. After depicting the thickness profile in three dimensions, the asymmetry of the thickness profile becomes even more pronounced, as shown in Fig. 8 ▸(*b*). The 2D profile from a section of the thickness profile taken along the horizontal diameter direction in Fig. 8 ▸(*a*) confirms the above analysis. The results are shown in Fig. 8 ▸(*c*), with the profile of an ideal lens provided for comparison. According to Fig. 8 ▸(*c*), the apical offset of the lens is 12.56 µm and the tilt angle is 14.01°, which will result in a severe deviation of the focal spot of the lens. Therefore, the measurement results can be used to guide the printing process optimization for lens additive manufacturing. Certainly, the DLD-STXI method can also be used to measure the manufacturing accuracy of other low-*Z* material devices precisely and efficiently.

## 4. Conclusion and discussion

This paper introduces a thickness profile metrology technique utilizing a single planar X-ray projection, based on precise phase retrieval through X-ray speckle tracking imaging enhanced by dark-field imaging. The method was validated with accuracy calibration using a uniform sample, achieving an RMSE of 1.033 µm with a detector effective pixel size of 0.65 µm. Subsequently, the technique’s capability to measure samples with arbitrary thickness profiles was demonstrated using a worn-out fiber. Additionally, an application involving a microlens array illustrated the method’s effectiveness in thickness metrology and associated manufacturing error analysis. Compared with CT, which typically requires a long time to acquire the numerous projections and reconstruct the 3D image, our method completes the measurement in just a few seconds with a single projection. This significant time efficiency makes it highly suitable for high-throughput inspection. Additionally, for samples with a laminar shape and large size – which are difficult to measure by CT – our projection-based technique offers a practical alternative.

For this imaging-based metrology technique, both the precision and the FOV are adjustable based on specific requirements. Allowing for a tolerance in the precision enables a broader measurement area, while a smaller FOV with higher magnification yields greater precision. In this study, a detector with an effective pixel size of 0.65 µm provided an FOV width of 1.33 cm, which extended to 4.71 cm with an effective pixel size of 2.3 µm. Larger array detectors could expand the measurement space further. However, this adaptability presents challenges in precision calibration due to variations in detector pixel size and other parameters that require corresponding adjustments, thereby influencing metrology errors. Current analysis suggests that measurement errors generally remain within twice the effective pixel size, offering a preliminary estimate of error based on pixel size. Future research will explore additional factors affecting measurement errors and assess the adequacy of the measurement accuracy.

In summary, our proposed metrology technique achieves *in situ*, non-contact, high-precision, large-FOV and efficient measurements through a single X-ray projection, making it particularly suitable for samples with complex geometries. The simplicity of the setup allows for seamless integration into industrial production lines. For example, a conveyor belt can be positioned within the optical path, enabling real-time non-contact quality inspection of components during manufacturing. This makes our method especially attractive for inline metrology and defect detection, where conventional CT systems are often too bulky, slow or complex to deploy effectively. In addition, this configuration facilitates the integration of *in situ* loading devices along the optical path. For instance, this method enables measurement of dynamic thickness changes during stretching or heating within experimental setups. Its application in production lines or additive manufacturing processes could enable real-time thickness inspection. We anticipate broad applicability of this measurement method in various complex scenarios.

## Figures and Tables

**Figure 9:**
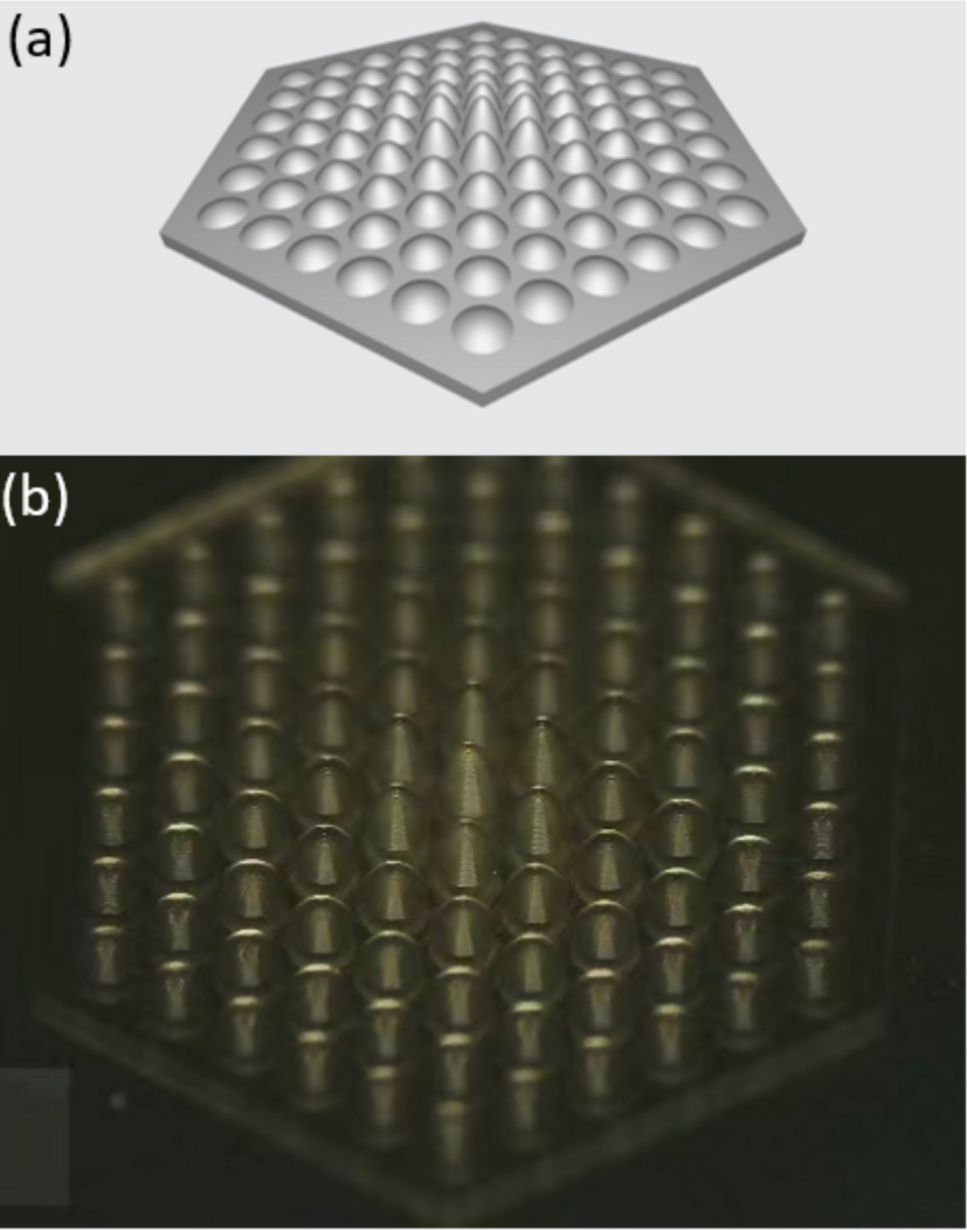
The multi-focal microlens array. (*a*) Design diagram and (*b*) microscopy image.

## Data Availability

The data that support the findings of this work are available from the corresponding author upon reasonable request.

## Appendix A IL-PCI CT

For a sample with a complex refractive index *n* = α + *i*β, IL-PCI is implemented by placing the sample into a monochromatic synchrotron X-ray beam, with a detector positioned downstream and aligned perpendicular to the beam path. The light intensity distribution  at a detector located a distance *z* from the sample is mathematically related to the phase shift distribution  caused by the sample (Gureyev *et al.*, 2004 ▸). This relationship can be described using the phase-attenuation duality (PAD) algorithm (Chen *et al.*, 2013 ▸),

where ɛ is the ratio of the real part α to the imaginary part β of the complex refractive index, and ξ and η are the spatial frequencies in Fourier space corresponding to a point (*x*, *y*) in real space.

Filtered back-projection was used to reconstruct the 3D CT slices from these phase-retrieved images (Burvall *et al.*, 2011 ▸),

where ν is the CT reconstruction filter and * denotes a one-dimensional convolution.

For single-material samples, where ɛ remains constant throughout, this method allows precise reconstruction of the internal 3D structure. Consequently, the thickness profile of the sample can be accurately derived from the reconstructed volume.

## Appendix B Multi-focal microlens array

The lens is mounted on a hexagonal base with a thickness of 100 µm. It was fabricated using projection micro-stereolithography 3D printing (nanoArch S130, BMF Precision Technology Co., China). Fig. 9 ▸(*a*) shows the design diagram. The sub-lens surface features a quadratic paraboloid shape with gradient variation parameters. It is evident that the central sub-lens is the highest, which corresponds to the shortest focal length. As one moves outwards, the sub-lens height decreases, leading to longer focal lengths. This configuration enables the device to achieve a broader depth of field. The design and fabrication approach were proposed by Dr Zhang’s group (Long *et al.*, 2021 ▸). Fig. 9 ▸(*b*) depicts a microscopy image of the completed product.
